# PIBS: Proton and ion beam spectroscopy for *in vivo* measurements of oxygen, carbon, and calcium concentrations in the human body

**DOI:** 10.1038/s41598-020-63215-0

**Published:** 2020-04-24

**Authors:** Paulo Magalhaes Martins, Riccardo Dal Bello, Benjamin Ackermann, Stephan Brons, German Hermann, Thomas Kihm, Joao Seco

**Affiliations:** 1 German Cancer Research Center – DKFZ, Heidelberg, Germany; 2 Instituto de Biofísica e Engenharia Biomédica, Faculdade de Ciências da Universidade de Lisboa, Lisboa, Portugal; 3 Department of Physics and Astronomy, University of Heidelberg, Heidelberg, Germany; 4 Heidelberg Ion-Beam Therapy Center, Heidelberg, Germany; 5 Max Planck Institute for Nuclear Physics, Heidelberg, Germany

**Keywords:** Metastasis, Cancer, Applied physics, Techniques and instrumentation

## Abstract

Proton and ion beam therapy has proven to benefit tumour control with lower side-effects, mostly in paediatrics. Here we demonstrate a feasible technique for proton and ion beam spectroscopy (PIBS) capable of determining the elemental compositions of the irradiated tissues during particle therapy. This follows the developments in prompt gamma imaging for online range verification and the inheritance from prompt gamma neutron activation analysis. Samples of water solutions were prepared to emulate varying oxygen and carbon concentrations. The irradiation of those samples and other tissue surrogate inserts by protons and ion beams under clinical conditions clearly showed a logarithmic relationship between the target elemental composition and the prompt gamma production. This finding is in line with the known logarithmic dependence of the pH with the proton molar concentration. Elemental concentration changes of 1% for calcium and 2% for oxygen in adipose, brain, breast, liver, muscle and bone-related tissue surrogates were clearly identified. Real-time *in vivo* measurements of oxygen, carbon and calcium concentrations will be evaluated in a pre-clinical and clinical environment. This technique should have an important impact in the assessment of tumour hypoxia over the course of several treatment fractions and the tracking of calcifications in brain metastases.

## Introduction

The analysis of the elemental compositions of the human body by means of irradiated particles was first described by Anderson *et al*. in 1964^1,2^. That technique was coined as *in vivo* neutron activation analysis^1^. That breakthrough gave rise to the measurements of total body hydrogen^3,4^, carbon^5^, nitrogen^6^, calcium and sodium^7,8^, and localized measurements of chlorine^9^ and cadmium^6,10^. The activated elements were identified because of their decay at different rates or because they emitted gamma-rays of different energy^1^. The latter was further developed and nominated as prompt gamma *in vivo* neutron analysis (PGIVNA or IVNAA). The evaluation of total body protein by IVNAA^11^ had a major impact in intensive care patients^12^, thus saving several billions of dollars annually round the World^13^. A comprehensive review of *in vivo* experimental methods to determine the composition of the human body has been carried out by Sutcliffe^14^ and Ellis & Eastman^15^.

The IVNAA relied either on high energy resolution germanium detectors (HPGe) for tracking calcium, cadmium and chlorine or by thick sodium iodide NaI(Tl) detectors for determining total body hydrogen and nitrogen. This technique was developed close to nuclear reactors, particle accelerators or radioisotope neutron sources. Many facilities, such as the ones at Birmingham^16^ and Brookhaven^4,17^, were fully designed with the purpose of studying the prompt activation for the spectroscopy of neutron activated elements. More recently, new techniques have emerged for the detection of heavier elements in human organs^18–20^. However, neutron therapy, although popular in the 70 s and 80 s, still offers low margin of benefit if compared to conventional photon therapy. By 2015, only 30,000 patients were treated with neutron therapy, mostly malignant melanoma and salivary glands tumours^21^. Nowadays, prompt gamma neutron activation analysis is mostly used in material science as an efficient non-destructive multi-elemental detection technique^22–24^.

Conversely, proton and ion-beam therapy is steadily expanding far and wide with approximately 200,000 patients already treated^25^. The number of facilities is also increasing rapidly with 100 centres currently in operation in 20 countries, 43 under construction in 7 new countries, and 24 centres planned in 4 new countries. In the era of precision medicine, radiation oncology is playing a decisive role to fight cancer^26,27^. Proton and carbon ion therapy constitutes a new landmark in the present times with vast clinical applications^28^. The research is expanding to other medical fields, such as cardiology^29^. There is also an increasing interest in understanding the radiobiological effects in living beings during space missions^30^.

In 2011, Polf *et al*. proposed to measure the prompt gamma ray emission during proton radiotherapy to assess treatment delivery and patient response^31^. A simulation from a proton beam with 250 MeV impinging on a hypoxic region with the oxygen concentration artificially changed from 70% to 50% clearly showed a logarithmic trend between the oxygen concentration and the production of proton-induced prompt gamma rays. In 2013, the same group demonstrated experimentally a linear relationship between the grams of irradiated oxygen in tissue-equivalent samples and the total emission of 6.13 MeV prompt gammas detected with a HPGe detector during irradiation by protons^32^. Recent work from the NIST group at the PGAA facility at the NIST Center for Neutron Research was able to determine with a Compton camera the hydrogen concentration from prompt-gamma emission during neutron irradiation^33^.

Prompt gamma imaging for real-time *in vivo* verification of the range of protons and ion beams in human tissue has been first proposed by Stichelbaut & Jongen^34^. Since then, several prototypes were developed^35–39^ and two systems already reached the clinical phase^40,41^. Prompt gamma spectroscopy (PGS) emerged as a technique that was able to measure absolute range deviations in the patient^37^. The Compton imaging has also shown the ability to determine the beam range and range shifts with 3D images^39^. Other systems that track range deviations between radiotherapy fractions will not allow online real-time adaptation of the treatment plan. The spectroscopic nature of PGS also allows for the determination of the elemental concentrations within the irradiated human tissues. This feature is linked to the particle range in the patient as the body composition will ultimately affect the location where the particles will stop, thus influencing the spatial resolution.

We describe the implementation of a new generation of scintillation detectors used for range verification that can measure the whole spectrum of ion-beam induced prompt gamma rays^42,43^. They allow simultaneous beam tracking and determination of elemental composition of tissue surrogates. We reproduced the results from Polf *et al*. for proton beams^32^ and extended it to helium and carbon beams. We also expanded the concept to a larger number of tissue surrogate inserts with varying concentrations of oxygen, carbon and calcium. A realistic scenario with clinical beam intensities and acquisition times was also considered. We demonstrated the ability to track 1% calcium variations between adipose and breast surrogates and 2% oxygen variations between the several tissue surrogates. We also demonstrated a logarithmic trend between the elemental concentration of the target and the prompt gamma production induced by the irradiation of such elements. Finally, we were able to track 3% oxygen concentration variations in the samples of water plus sugar after 7 cm depth in water.

## Results

**Figure 1:**
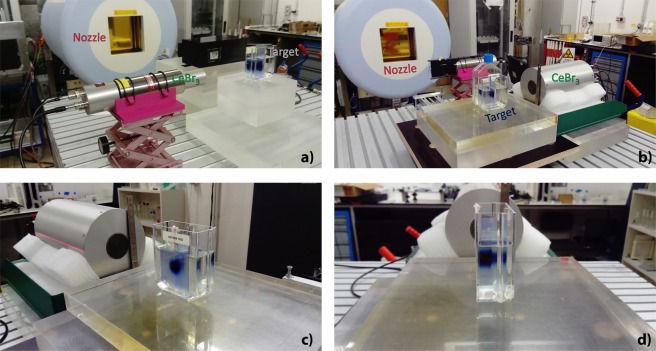
Experimental setup. (**a**) Photo of the experimental setup consisting of an irradiating nozzle, a small target, and a CeBr_3_ detector. (**b**) Photo of a similar setup with a larger target irradiated with a higher energy. (**c**) Photo of the frontal face of the target with an EBT film placed perpendicular to a sharp oxygen beam. (**d**) Photo of the lateral view of the same target with an EBT film placed within the target and along the beam axis.

We started by irradiating the samples of water plus sugar with proton beams with the lowest energy available and confirmed the results obtained by Polf *et al*.^32^. Afterwards, we increased the energy of our beam to a clinically relevant energy. Figure 1a,b show the detector, the target and the beam nozzle. Figure 1c shows a dosimetric film placed in the frontal part of the target and perpendicular to a sharp oxygen beam. Figure 1d shows another dosimetric film placed longitudinally within the sample and along the beam axis.

**Figure 2:**
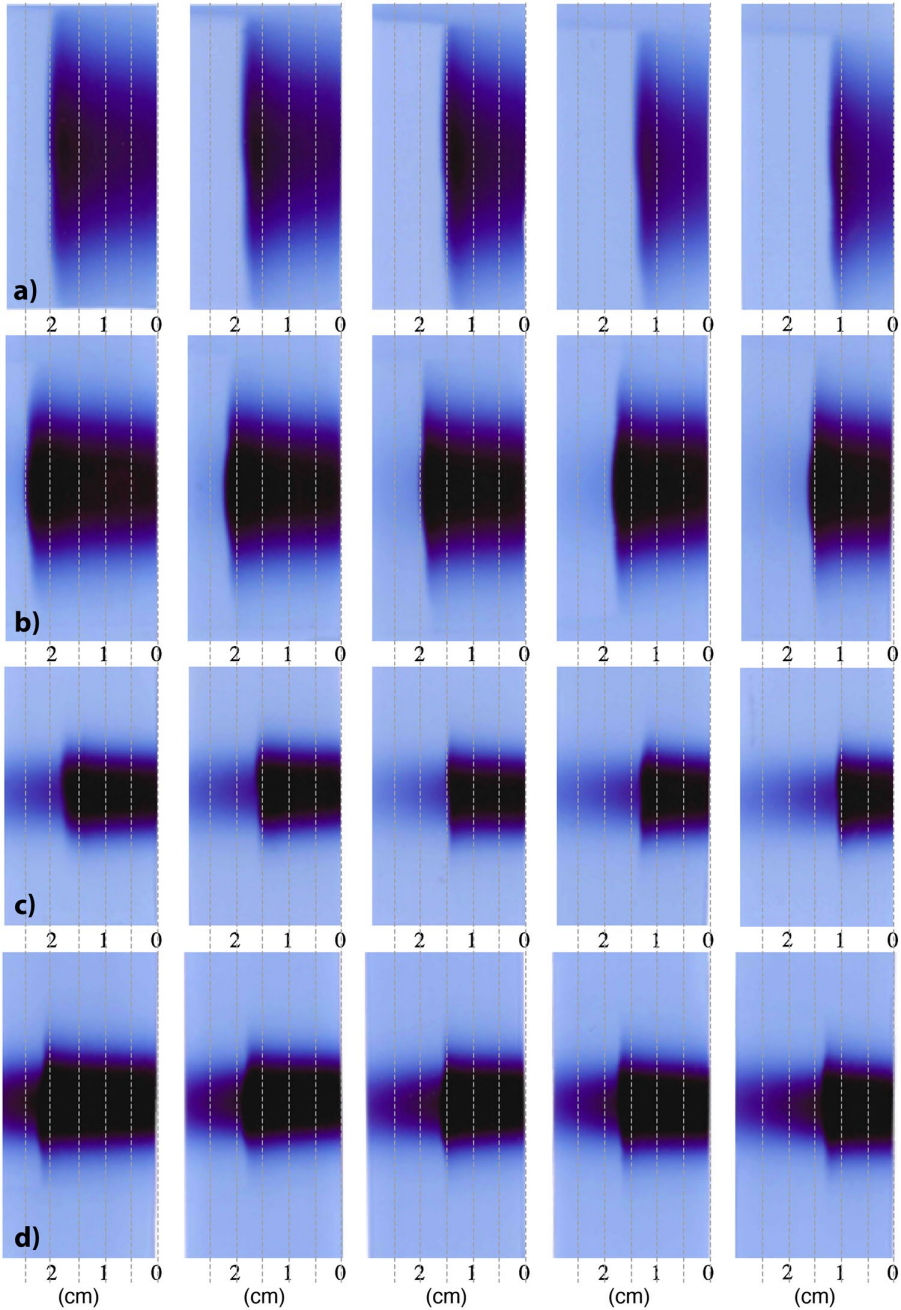
EBT films irradiated by protons and ions of helium, carbon and oxygen. The five films in each row are the ones placed longitudinally within the sample and along the beam axis. They are ordered for an increasing density. The higher the density the shorter the path travelled within the sample. The irradiated distance = EBT irradiated film (cm) + 3.8 cm (first flask). (**a**) Film irradiated by a proton beam. A large penumbra and a very steep dose fall-off are observed. (**b**) Film irradiated by a helium beam. Lateral scattering is less evident, but a less steep dose fall-off is observed. (**c**) Film irradiated by a carbon beam. The spot is much sharper, but a considerable fragmentation after the stopping point is observed. (**d**) Film irradiated by an oxygen beam. The spot is very sharp, but at the cost of a very strong fragmentation after the stopping point.

In Fig. 2, we show the films irradiated from the right side by protons and ions of helium, carbon and oxygen. The higher the density of the sample, the shorter the travelled distance within the sample. Also, the heavier the ion, the sharper the spot. We observe that for heavier ions, there is an increasing fragment component after the stopping point. Table 1 summarizes the irradiated volumes and the corresponding mass of oxygen irradiated for five samples. We observe that for the same energy steps the volume irradiated for protons ranges from 12.2 cm^3^ to 14 cm^3^. For helium, carbon and oxygen beams the irradiated volume is reduced by 63%, 89% and 92%, respectively. These ratios depend on the spot size as the travelled distance is kept essentially the same for the four beam species after selecting the proper initial energies.

**Table 1:** Volume and mass of oxygen irradiated by proton, helium, carbon, and oxygen beams impinging on five samples of water plus sugar.

Sample			Water 130 g	+25 g sucrose	+50 g sucrose	+75 g sucrose	+130 g sucrose
**Protons**	Energy	$\rho$ (g cm^−3^)	1	1.04	1.09	1.14	1.21
Spot size 17.4 mm	90.7 MeV	V (cm^3^)	14	13.6	12.9	12.4	12.2
		${\text{m}}_{{O}_{2}}$ (g)	12.5	11.7	11.1	10.6	10.3
**Helium**	Energy	$\rho$ (g cm^−3^)	1	1.04	1.09	1.14	1.21
Spot size 10.2 mm	92 MeV/u	V (cm^3^)	5.19	4.94	4.78	4.64	4.41
		${\text{m}}_{{O}_{2}}$ (g)	4.61	4.26	4.08	3.97	3.74
**Carbon**	Energy	$\rho$ (g cm^−3^)	1	1.04	1.09	1.14	1.21
Spot size 5.9 mm	169.2 MeV/u	V (cm^3^)	1.56	1.50	1.46	1.41	1.35
		${\text{m}}_{{O}_{2}}$ (g)	1.39	1.29	1.25	1.21	1.15
**Oxygen**	Energy	$\rho$ (g cm^−3^)	1	1.04	1.09	1.14	1.21
Spot size 4.9 mm	119.2 MeV/u	V (cm^3^)	1.15	1.08	1.05	1.03	0.98
		${\text{m}}_{{O}_{2}}$ (g)	1.03	0.93	0.90	0.88	0.83

**Figure 3:**
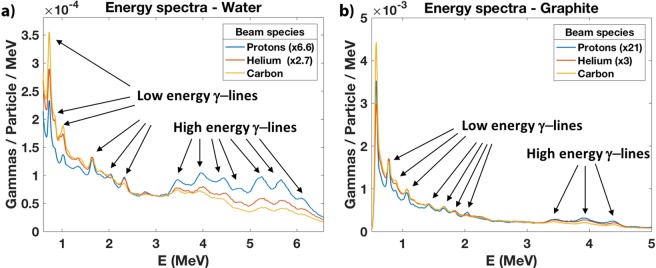
Energy spectra obtained from the irradiation of water and graphite by protons, helium, and carbon beams. (**a**) The low energy $\gamma$-lines, particularly the 0.718-MeV carbon line, show a higher prominence for carbon beams, while the high energy $\gamma$-lines show a higher prominence for proton and helium beams. (**b**) The carbon induced $\gamma$-yield is about 21 times higher than protons.

Figure 3 shows the prompt gamma energy spectra from the irradiation of water and graphite by protons, helium, and carbon beams. We observe that protons and helium ions produce a higher prompt-gamma yield above 3 MeV, while carbon ions produce a higher prompt-gamma yield below 3 MeV, particularly at 0.718 MeV. Therefore, protons and helium ions are better suited to measure high energy prompt gammas and carbon ions better suited to measure low energy prompt gammas. We also observe that the carbon ions produce prompt gamma yields about 6.6 and 21 times higher than protons for the water and graphite targets, respectively.

**Figure 4:**
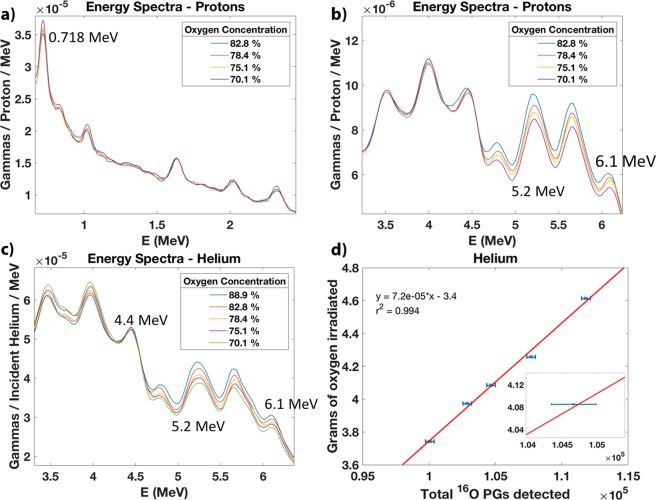
Energy spectra resulting from the irradiation of samples with varying oxygen concentrations by protons and helium beams. (**a**) The low energy region shows an energy line at 0.718 MeV that increases with an increasing carbon concentration or decreasing oxygen concentration. (**b**) The high energy region shows two energy lines at 5.2 and 6.1 MeV that increase with an increasing oxygen concentration. (**c**) The helium beams also present the same behaviour for the 5.2 and 6.1 MeV lines. Conversely, the single- and double-escape peaks from the 4.4 MeV lines decrease with an increasing oxygen concentration. (**d**) The total number of prompt gammas (PGs) detected within the 5.2 MeV peak resulting from the irradiation of the five samples by helium beams has a linear relationship with the measured grams of oxygen irradiated. The inset shows the random error associated to the counts in the 5.2 MeV peak. The average uncertainty on the irradiated mass is Δ${m}_{O}=46.7\,\text{mg}$.

We present the energy spectra from the irradiation of the samples of water plus sugar for the low energy prompt gammas (Fig. 4a) and for the high energy prompt gammas (Fig. 4b). We observe that at 0.718 MeV, there is a decreasing prompt-gamma production with an increasing oxygen concentration. This energy line results from the excitation of carbon nuclei followed by the prompt emission with 0.718 MeV. Conversely, the 5.2 MeV and the 6.1 MeV energy peaks increase with an increasing oxygen concentration. These energy lines result from the excitation of oxygen nuclei followed by the prompt emission with the corresponding energy. The same behaviour is observed for the same samples irradiated by helium ions for the high energy prompt gamma region (Fig. 4c). Moreover, we observe that the 4.4 MeV line remains unchanged because of competing reactions with oxygen and carbon nuclei. However, the corresponding single- (3.9 MeV) and double-escape peaks (3.4 MeV) decrease with an increasing oxygen concentration. After determining the mass of oxygen irradiated for every sample, we plotted a linear trend between the grams of oxygen irradiated by helium and the total of prompt gammas detected within the 5.2 MeV energy peak (Fig. 4d). The average statistical uncertainty on the irradiated mass is Δ${m}_{O}=46.7\,\text{mg}$ (see Supplementary Fig. S1). Approximately 2.6 million counts were recorded for each run.

**Figure 5:**
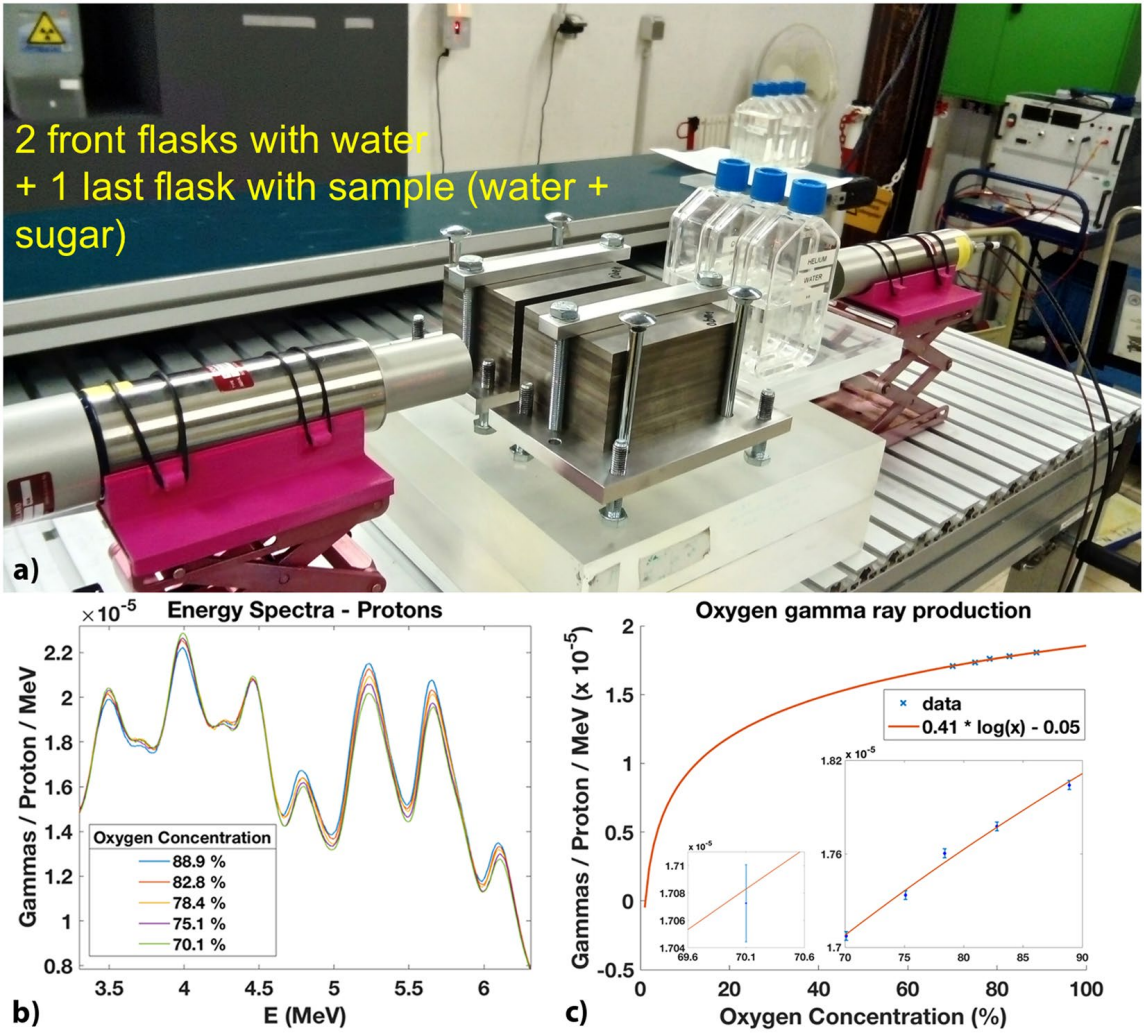
Tracking varying oxygen concentrations after 7 cm depth in water. (**a**) Photo of the experimental setup with two front flasks of water and a third flask with the sample of water plus sugar. Only the uncollimated detector was active during the acquisition. (**b**) Energy spectra from the irradiation of the five samples of water plus sugar by proton beams. There is a clear increase of the prompt gamma production for the 5.2 and 6.1 MeV peaks with an increasing oxygen concentration. (**c**) The data obtained fit a logarithmic trend between the oxygen concentration and the prompt gamma production. The insets show the random error associated to the counts in the 5.2 MeV peak, particularly for an oxygen concentration of 70.1%. The average statistical uncertainty is 2.85 × 10^−8^. The uncertainty in the oxygen concentration ranges from Δ$[O]=0.95 \%$ ([O] = 70.1%) to Δ$[O]=1.24 \%$ ([O] = 88.9%).

We aimed at reproducing the previous results with the five samples of water plus sugar placed at a 7 cm depth after water, i.e. just behind two water flasks. Figure 5a shows a setup where our target is composed by two flasks of water and the third flask contains one of the prepared five samples. Figure 5b shows the energy spectra resulting from the irradiation of the three flasks by protons. Only the last flask was changed between irradiations. Also here, we observe a clear trend between the increase in oxygen concentration and the prompt gamma production with an energy of 5.2 MeV and 6.1 MeV. The few data points obtained for high oxygen concentrations fit within a logarithmic trend between the oxygen concentrations and the prompt gamma production (Fig. 5c). The average statistical uncertainty is 2.85 × 10^−8^ (see Supplementary Fig. S2). The uncertainty in the oxygen concentration ranges from Δ$[O]=0.95 \%$ ([O] = 70.1%) to Δ$[O]=1.24 \%$ ([O] = 88.9%). We recorded between 7.5 and 8 million counts for each run.

**Figure 6:**
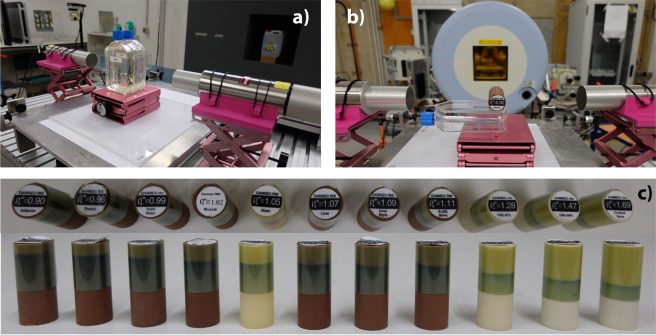
Experimental setup to compare the oxygen and calcium concentration from the samples of water plus sugar and the tissue surrogate inserts. (**a**) Photo of the sample of water plus 180 g of sugar to be irradiated by helium beams with 88.1 MeV/u. (**b**) Photo of the adipose surrogate insert to be irradiated by a helium beam with the same energy. (**c**) Photo of the top and side view of the tissue surrogate inserts wrapped in EBT film. The density of the inserts increase from the left to the right. The beam coming from the bottom travels a shorter path length for denser materials.

In the last setup, we compared the results from the irradiation of the samples of water plus sugar with the results from the irradiation of the tissue surrogate inserts. Figure 6a shows the sample of water plus 180 g of sugar to be irradiated by helium ions. Figure 6b shows the adipose surrogate insert to be irradiated by helium ions as well. Both the samples and the inserts were placed with the expected point of maximum dose deposition aligned with the detectors. Figure 6c shows the tissue surrogate inserts with EBT films wrapped around them. Supplementary Fig. S3 shows four exemplary tissue surrogates inserts (adipose, brain, PMMA and cortical bone) wrapped in EBT film. We observe in both figures that the higher the density the shorter the path travelled by the beam within the insert. The beam is irradiated through the axis of the cylinder. Therefore, only the penumbra reaching the film is visible. The films ordered according to the density of the tissue surrogates for a helium beam coming from the left are presented in Supplementary Fig. S4. A profile along them shows the steep fall-off of the dose after the stopping point (see Supplementary Fig. S5). This distance was used to determine the irradiated volume.

**Figure 7:**
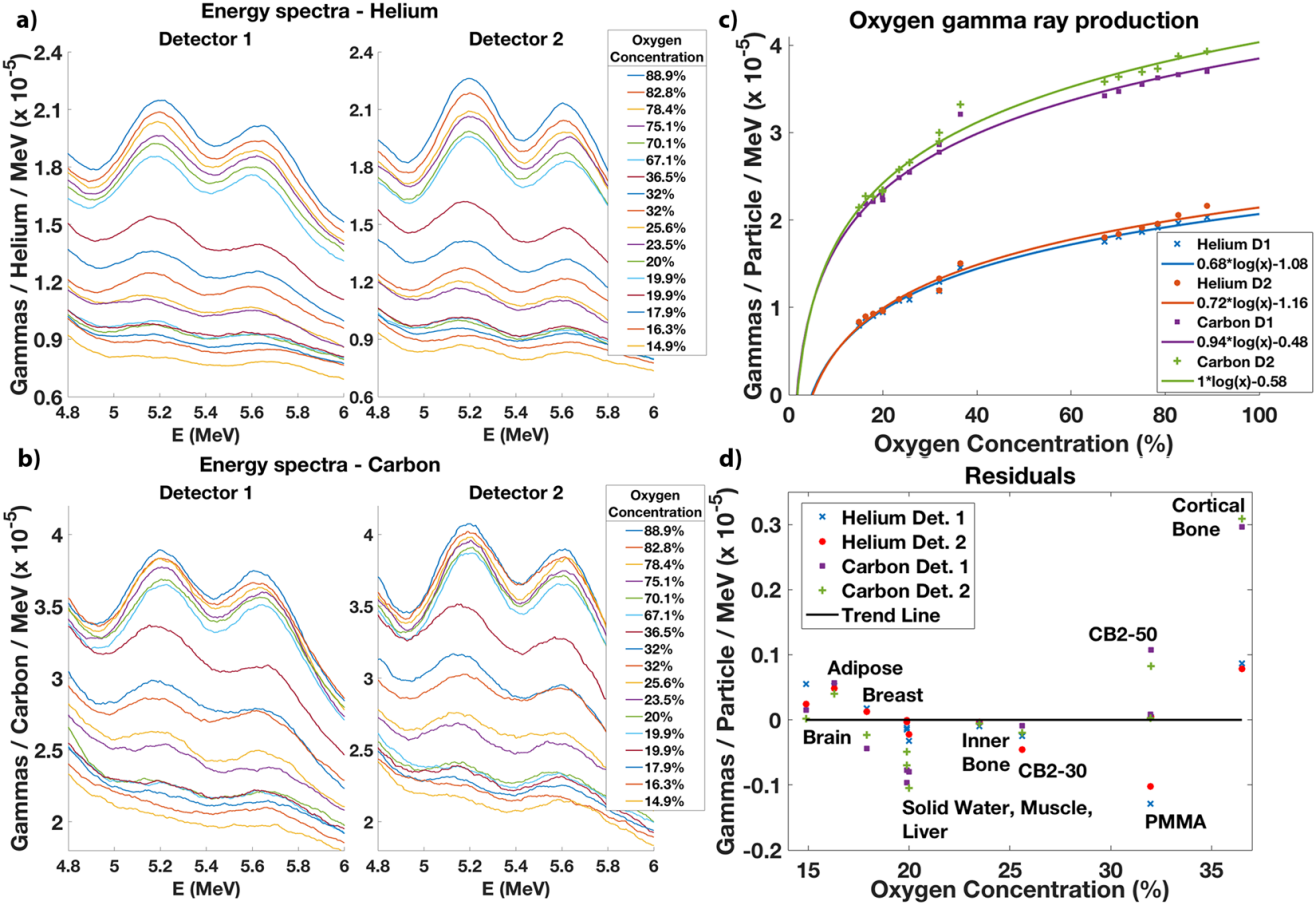
Relationship between the oxygen concentration and the prompt gamma production. (**a**,**b**) Prompt gamma energy spectra resulting from the irradiation of 6 water solutions and 11 tissue surrogate inserts by helium and carbon beams. (**c**) The data from the irradiation of the samples of water plus sugar with higher oxygen concentration and the data from the irradiation of the tissue surrogate inserts with lower oxygen concentration clearly fits a logarithmic trend. (**d**) The residuals between the data points and the trend for each tissue surrogate show small deviations for both beam species and both detectors. Results from brain, adipose, CB2-50 and cortical bone surrogates are over the trend, while the ones from solid water, muscle, liver, inner bone and CB2-30 surrogates are below the trend. Results from PMMA are below the trend for helium beams, and the ones from CB2-50 surrogate are over the trend for carbon beams. The average statistical uncertainty is 5.34 × 10^−8^ for helium ions and 7.88 × 10^−8^ for carbon ions. The uncertainty in the oxygen concentration ranges from Δ$[O]=0.20 \%$ ([O] = 14.9%) to Δ$[O]=1.83 \%$ ([O] = 88.9%) for helium ions and from Δ$[O]=0.22 \%$ ([O] = 14.9%) to Δ$[O]=1.55 \%$ ([O] = 88.9%) for carbon ions.

After irradiating the water solutions and the tissue surrogate inserts, we obtained a prompt gamma energy spectrum for each target. Figure 7a,b show the 17 prompt gamma spectra for the high energy region obtained after the irradiation by helium and carbon beams, respectively. We also show the results from 2 independent detectors facing each other at a similar distance to the target. We observed that the higher the oxygen concentration of the target the higher the prompt gamma production within the 5.2 MeV energy peak. These results close the gap between the low oxygen concentrations from the tissue surrogate inserts and the high oxygen concentrations from the samples of water plus sugar. The results shown in Fig. 7c clearly match a logarithmic trend as simulated by Polf *et al*.^31^. The prompt gamma production is given by *k*_1_ × *log*(*x*) − *k*_2_, where $x$ is the oxygen concentration, and ${k}_{1}$ and ${k}_{2}$ are constants depending on the geometry (e.g., distance from the detector to the target) and the beam species. Figure 7d shows the residuals between the data points and the trends. The deviations from the trend are about 5 × 10^−7^ which compares with a statistical error one order of magnitude lower. The brain and adipose surrogates present slightly higher values, and the cortical bone surrogate is more overestimated than the values given in Table 2. Conversely, solid water, muscle, liver, inner bone and CB2-30 surrogates are slightly underestimated than the values given in Table 2. The CB2-50 surrogate presents higher values for carbon beams, while PMMA presents lower values for helium beams. Breast surrogate shows slightly higher values for helium beams and lower values for carbon beams. The average statistical uncertainty is 5.34 × 10^−8^ for helium ions and 7.88 × 10^−8^ for carbon ions (see Supplementary Fig. S6). The uncertainty in the oxygen concentration ranges from Δ$[O]=0.20 \%$ ([O] = 14.9%) to Δ$[O]=1.83 \%$ ([O] = 88.9%) for helium ions and from Δ$[O]=0.22 \%$ ([O] = 14.9%) to Δ$[O]=1.55 \%$ ([O] = 88.9%) for carbon ions (see Supplementary Fig. S8). We recorded from 1.7 × 10^6^ counts (21 spills of helium ions hitting the liver surrogate) to 2.9 × 10^6^ counts (28 helium beam spills hitting the water solution with 130 g of sugar). For carbon ions, we recorded from 1.6 × 10^6^ counts (21 beam spills hitting the brain surrogate) to 2.6 × 10^6^ counts (28 beam spills hitting the same water solution). If we consider only 7 spills, which corresponds to 33.93 s of irradiation, the uncertainty in the oxygen concentration ranges from Δ$[O]=0.36 \%$ ([O] = 14.9%) to Δ$[O]=3.4 \%$ ([O] = 88.9%) (see Supplementary Fig. S9). In the case of a single spill lasting 4.84 s, the uncertainty in the oxygen concentration ranges from Δ$[O]=0.97 \%$ ([O] = 14.9%) to Δ$[O]=9.2 \%$ ([O] = 88.9%) (see Supplementary Fig. S10).

**Table 2:** Density and elemental compositions (% by mass) of six samples of water plus sugar and 11 tissue surrogate inserts.

		Density (g cm^−3^)	Composition (% by mass)			
			Oxygen	Carbon	Hydrogen	Calcium
Sample	Water	1	88.9	0	11.1	0
	+25 g sucrose	1.04	82.8	6.8	10.4	0
	+50 g sucrose	1.09	78.4	11.7	9.9	0
	+75 g sucrose	1.14	75.1	15.4	9.5	0
	+130 g sucrose	1.21	70.1	21.1	8.8	0
	+180 g sucrose	1.35	67.1	24.4	8.5	0
Tissue surrogate	Adipose	0.943	16.3	72.3	9.1	0
	BR-12-Breast	0.983	17.9	70.1	8.6	0.95
	Solid-Water	1.015	19.9	67.3	8	2.31
	Muscle-Gammex	1.053	19.9	67.2	8.1	2.32
	BRN-SR2-Brain	1.053	14.9	72.5	10.8	0
	LV1-Liver	1.094	20	67	8.1	2.31
	IB-Inner-Bone	1.129	23.5	55.6	6.7	8.86
	PMMA	1.180	32	60	8	0
	CB2-30	1.334	25.6	53.5	6.7	12.01
	CB2-50	1.559	32	41.6	4.8	20.01
	SB3-Cortical-Bone	1.821	36.5	31.4	3.4	26.81

**Figure 8:**
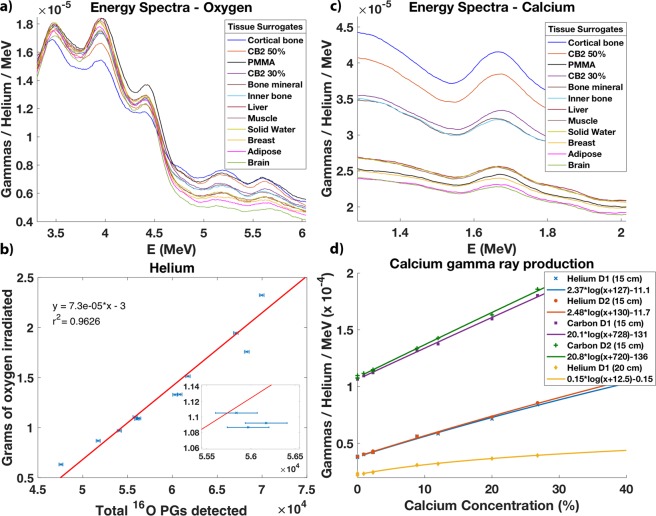
Relationship between the elemental concentration, the mass irradiated and the prompt gamma production. (**a**) Prompt gamma energy spectra resulting from the irradiation of 12 tissue surrogate inserts by helium beams. A windowing over the region where oxygen and carbon reactions predominate is shown. (**b**) The measured grams of oxygen irradiated in the tissue surrogate inserts and the total prompt gammas (PGs) detected within the 5.2 MeV peak are linearly related. The linear fit excluded the PMMA outlier. The average uncertainty in the irradiated oxygen mass is Δ${m}_{O}=35.6\,\text{mg}$. (**c**) A windowing over the 1.66 MeV energy line shows clear differences in prompt gamma production attributable to calcium reactions. (**d**) The data points relating the calcium concentrations and the prompt gamma production from the irradiation of the tissue surrogates by helium and carbon beams are shown for two detectors and one detector placed at 15 cm and 20 cm from the beam axis, respectively. The minima for calcium correspond to adipose and brain surrogates. It is clearly distinguishable from breast surrogate (0.95%) and from solid water, muscle and liver surrogates (2.3%). The maximum concentration appears in cortical bone. The average statistical uncertainty is 1.72 × 10^−7^ for helium ions and 2.74 × 10^−7^ for carbon ions. The uncertainty in the calcium concentration ranges from Δ$[Ca]=0.14 \%$ ([Ca] = 0.95%) to Δ$[Ca]=0.28 \%$ ([Ca] = 26.8%) for helium ions and the detector at 15 cm. For carbon ions, it ranges from Δ$[Ca]=0.15 \%$ ([Ca] = 2.31%) to Δ$[Ca]=0.25 \%$ ([Ca] = 26.8%).

Finally, we evaluated if the relationship between the elemental concentration and the total prompt gammas detected also holds true for other elements, such as calcium. Here, we irradiated just the tissue surrogate inserts by helium beams after wrapping them with EBT film. Figure 8a shows 12 prompt gamma spectra for the high energy region. We observed again that the higher the oxygen concentration of the tissue surrogate the higher the prompt gamma production within the 5.2 MeV energy peak. Conversely, the higher the carbon concentration of the tissue surrogate the higher the prompt gamma production within the 4.4 MeV single and double-escape energy peaks. Figure 8b confirms and reproduces the linear relationship between the grams of oxygen irradiated by helium and the total prompt gammas produced within the 5.2 MeV peak both for the water solutions and the tissue surrogate inserts: *m*_*O*_[g] = 7.3 × 10^−5^ × ${N}_{{\gamma }_{5.2MeV}}$ − 3. The average uncertainty in the irradiated mass is Δ${m}_{O}=35.6\,\text{mg}$ (see Supplementary Fig. S7). Similar results are reproduced for helium and carbon beams with two detectors placed at 15 cm from the beam axis (see Supplementary Fig. S13). Figure 8c shows the prompt gamma energy spectra resulting from the irradiation of the tissue surrogate inserts by helium within a low energy window. The peaks observed result from the irradiation of calcium followed by the emission of prompt gamma with 1.66 MeV. At the same energy, there is a competing reaction. We observe an increasing energy line for the 6 water solutions with an increasing carbon concentration or decreasing oxygen concentration (see Supplementary Fig. S14). However, since the cross-sections for carbon and oxygen are lower than the ones for calcium, they have little impact in the calcium line. Figure 8d) shows a clear relationship between the calcium concentrations and the prompt gamma production. Breast surrogate insert has a calcium concentration of 0.95%, while adipose and brain surrogate inserts have no calcium. Solid water, muscle and liver surrogate inserts have a concentration of 2.3%. Those variations are clearly distinguishable from the prompt gamma production. The average statistical uncertainty is 1.72 × 10^−7^ for helium ions and 2.74 × 10^−7^ for carbon ions (see Supplementary Fig. S6). The uncertainty in the calcium concentration ranges from Δ$[Ca]=0.14 \%$ ([Ca] = 0.95%) to Δ$[Ca]=0.28 \%$ ([Ca] = 26.8%) for helium ions and the detector at 15 cm. For carbon ions, it ranges from Δ$[Ca]=0.15 \%$ ([Ca] = 2.31%) to Δ$[Ca]=0.25 \%$ ([Ca] = 26.8%) (see Supplementary Fig. S11). We recorded from 2.5 × 10^6^ counts (14 helium beam spills hitting the brain surrogate) to 3.4 × 10^6^ counts (14 helium beam spills hitting the cortical bone surrogate). The difference in the helium spectra shows that a difference of 5 cm in distance from the detectors to the beam axis almost doubles the count rate. In the case of a single spill lasting 4.84 s, the uncertainty in the calcium concentration ranges from Δ$[Ca]=0.46 \%$ ([Ca] = 0.95%) to Δ$[Ca]=1.9 \%$ ([Ca] = 26.8%) (see Supplementary Fig. S12).

## Discussion

Prompt gamma spectroscopy is nowadays the most promising technique for range verification of protons and light ion beams in patients^41,43^. The spectroscopic nature of this technique also allows for the determination of the elemental compositions of the human body^1^. Its use in proton therapy centres for the assessment of treatment delivery and patient response was proposed^31^ and demonstrated either in non-clinical scenarios^32^ or as a free parameter in the context of range verification^37^. The former set the grounds for the present study.

In this paper, we present a clinical scenario where protons, helium and carbon ions irradiate not only samples of water plus sugar solutions with high oxygen concentration, but also tissue surrogate inserts with lower oxygen concentration and variable calcium concentrations. Conversely to previous works^32,44–46^, we used CeBr_3_ detectors instead of HPGe detectors. These detectors are at the moment more prepared for clinical conditions than HPGe detectors, both for the online range verification^42,43,47^ and the determination of the target elemental compositions.

In our first set of measurements, we confirmed the results obtained by Polf *et al*.^32^. We then observed that an increase in the target oxygen concentration also leads to an increase in the prompt gamma emitted from the oxygen irradiated by proton and helium beams with higher energies. By placing EBT films longitudinal to the beam, we could determine the volume irradiated and therefore calculate the mass of oxygen irradiated. As shown by Polf *et al*.^32^, the total prompt gammas emitted from the oxygen irradiated increases linearly with the mass of oxygen irradiated. Changes of 3% in oxygen concentration were also identified in samples of water solutions placed after 7 cm of water. These results confirm the ability to measure varying oxygen concentrations deeper in water phantoms.

In order to cover the low and high oxygen concentrations and demonstrate this technique for the whole concentration levels, 11 tissue surrogate inserts with oxygen concentrations ranging from 14.9% in the brain up to 36.5% in the cortical bone were irradiated. The results were compared with the ones obtained with the samples with high oxygen concentration. A logarithmic trend between the oxygen concentration in the target and the prompt gamma production both for helium and carbon beams was observed. A reproducible relationship between the mass of oxygen within the target irradiated by helium beams and the total prompt gammas with 5.2 MeV was verified.

Finally, a relationship between the calcium concentration and the prompt gammas produced from the calcium irradiation was evaluated. Variations of 1% in calcium concentration between breast surrogate and brain, and adipose surrogates (1.3% between the breast surrogate and muscle, solid water, and liver surrogates) produced significative differences in prompt gamma production.

The measurement of the elemental concentration in the irradiated tissues may play an important role in the treatment adaptation within fractionated plans. Nowadays, patients are daily irradiated with standard doses over several fractions (up to 30). If we could assess tumour response (such as changing hypoxia levels) between fractions without additional imaging modalities (such as CT, MRI, or PET) this could help the clinicians to better adapt treatment^48^. This would bring radiotherapy closer to personalized treatment.

Hypoxia is a characteristic feature of locally advanced solid tumours resulting from an imbalance between dioxygen (O_2_) supply and consumption^49^. One of the pathways leading to cell death is by indirect DNA damage due to the creation of free radicals and reactive oxygen species. In that case, dioxygen must be present for photons to cause DNA damage at the molecular level.

In this work, we evaluated only the water molecule oxygen. These measurements could provide indirect means for measuring the tissue perfusion and the changes in microvascular density that affects such perfusion^50^. Levels of hypoxia in cancer cells are known to affect the efficacy of conventional photon radiotherapy. There are many causative factors. Hypoxic cells are almost completely insensitive to such therapy and can be found in tumour areas with a poor blood supply. Particle therapy with carbon ions is more effective than conventional therapy in treating hypoxic tumours. Therefore, an inter-fractional assessment of the oxygen concentration of tumours irradiated by protons and light ions may provide also inputs for further research in this field.

It was already demonstrated that different tumours or tissue types have different elemental compositions^51^. Tumour samples were dried to determine the water content and the elemental compositions of the dried samples determined by combustion analysis. In proton and light ion beam therapy, one could possibly compare the elemental compositions obtained from PIBS and from combustion analysis. This would open new opportunities to determine the tumour composition and type without biopsy or excision. Prompt-gamma neutron activation analysis has already been implemented for *in vivo* body quantification of chlorine and nitrogen in small animals (rats and rabbits), which are considered to be indexes of extracellular fluid and protein, respectively^52^. Systematic measurements in mice are therefore needed to compare tumour elemental compositions obtained by means of prompt gamma measurements during irradiation and by combustion analysis of the tumour samples obtained by biopsy or at the time of excision.

Nuclear magnetic resonance spectroscopy provides unique features for the determination of the structure and dynamics of molecules. It is however just applicable to samples containing nucleus with spin, such as protons, carbon-13 and phosphorus-31. Some studies were able to relate the potentiometrically determined pH and the ^1^H NMR pH^53,54^ as well as the pH and the phosphorus-31 NMR^55^. The pH is given by the negative of the base 10 logarithm of the proton molar concentration. In this work, we present oxygen and calcium concentration trends from prompt gamma measurements that suggest a logarithmic relationship between the two. Several examples exist in different sciences, where the relationship between physical properties has been established on empirical observations, without any theoretical globalizing model. An example that comes close to the observations reported here is the kinetics of the chemical and/or nuclear reactions. All nuclear reactions are first order reactions, while chemical reactions can follow several orders that can also have fractional values. The determination of the order of the reaction is an important parameter in kinetics, which can only be determined experimentally, not being related to the stoichiometric coefficients. The order of a reaction is the power dependence of the rate of a reaction on the concentration or partial pressure of the reactant(s). In rate laws of order zero, the concentration of the reactant changes linearly with time. However, in rate laws of order one, it is the logarithm of the reactant concentration that changes linearly with time. The logarithmic dependence of the prompt gamma production on the oxygen, carbon, and calcium concentrations, not measurable by NMRS, will be further investigated with PIBS. There are also very recent advances for real-time monitoring of internal physiological processes by means of spectroscopic bioresorbable photonic devices that can also measure oxygenation levels and calcium concentration^56^.

Absolute measurements of total-body calcium using prompt gamma have been reported in renal patients^57^. Calcified brain metastases have been demonstrated by computed tomography^58^ and radiography^59^. However, the sensitivity of those techniques to minor calcification is low. Because the calcification usually occurs in a region of necrosis, significant enhancement in this necrotic region is not seen by CT. PIBS could be more sensitive to calcification in metastases. It could also support other imaging modalities, such as CT and MRI, to identify calcifying pseudoneoplasms of the neuraxis^60^.

## Methods

### Clinical application

In our experiments, we used CeBr_3_ detectors located at 15 and 20 cm from the beam axis. The acquisitions lasted between 1 and 3 min and both low and high clinical intensities were used (15–50 pA). The maximum total delivered particles were 4 × 10^10^ for protons and 2 × 10^10^ for helium ions. These conditions are close to the clinical standards.

This compares with the previous work from Polf *et al*.^32^, where the minimum energy available in proton centres was used at lower currents (7.5–15 pA). A germanium (HPGe) detector was located at a distance of 30 cm from the beam axis and the acquisitions lasted 20 min for a total 0.56–1.13 × 10^11^ irradiated protons. Four samples of water plus sugar with different elemental concentrations were prepared for irradiation.

### The HIT facility

The Heidelberg Ion-Beam Therapy Center - HIT^61^ accelerates proton, helium, carbon, and oxygen ions from 48 MeV/u up to 515 MeV/u. While protons and carbon ions are routinely implemented in the clinical setting, helium ions are currently being commissioned^62,63^, and oxygen ions still remain as a research beam species.

The intensities in clinical practice range from 2 × 10^6^ p/s for carbon ions to 3.2 × 10^9^ p/s for protons. There are two horizontal rooms and a fully 360° gantry for therapy. There is a horizontal experimental room where all the experiments referred in this paper were performed.

### Samples of water plus sugar and tissue surrogate inserts

We prepared samples of water plus sugar with different densities and elemental compositions. This choice aimed at reproducing the results from Polf *et al*.^32^. We prepared two extra samples with 50 g and 180 g of sugar. Table 2 shows the density and the elemental composition (% by mass) of the different samples. The considered water mass was 130 g.

We used the tissue surrogate inserts present in the Gammex phantom SN1612. Those are primarily used to measure the precision of the Hounsfield values in the CT datasets. Those values influence the dose distributions, in particular the calculated range. Table 2 shows the density and the elemental composition (% by mass) of the different tissue surrogate inserts. The results from Fig. 8 also include the bone mineral (B200) surrogate which is identical to the inner bone surrogate in composition and slightly different in density (${\rho }_{B200}$ = 1.146 g cm^−3^).

In total, we irradiated 18 different materials. From those, 6 were samples of water plus sugar with higher oxygen concentration and 12 were tissue surrogate inserts with lower oxygen concentrations. Five types of bone surrogates had an increased calcium concentration, while breast, solid-water, muscle and liver surrogates had very low calcium concentrations.

### Experimental setup

The main components of the experimental setup are the nozzle, the target, the EBT films and the CeBr_3_ detectors. These detectors are scintillation detectors with very good time and energy resolution. They are mainly used for range verification of the proton and ion beams in the patient. The CeBr_3_ detectors were aligned with the point in the target with the largest dose deposition and positioned at a distance of 15 cm from the beam axis (Fig. 6). For the results presented in Fig. 8, the detector  was placed at a distance of 20 cm from the beam axis. The CeBr_3_ crystals are identical in size (diameter d = 3.81 cm and length l = 7.62 cm). One crystal was coupled to a Hamamatsu R13089 photomultiplier tube (PMT) and the other one to a Hamamatsu R9420-100 PMT. Both detectors were plugged to a voltage divider. The anode output fed our data acquisition system (DAQ)^64^. This is a module of a FlashCam FADC system, originally designed for the Cherenkov Telescope Array (CTA)^65^.

### EBT films

For the samples of water plus sugar, the EBT films were placed within the target along the beam direction. Those films show at which position the beam stopped. This is essential to calculate the irradiated volume and the mass of oxygen irradiated. The irradiated volume was retrieved from the full width at half maximum (FWHM) of the spot size and the travelled distance, $L$, within the samples and the tissue surrogate inserts. We assumed that the irradiated volume is comprised within a cylinder of length $L$ and radius *r* = FWHM/2. The irradiated mass is related to the irradiated volume by $m=V\times \rho \times {F}_{0}$, where $m$ is the irradiated mass, $\rho$ is the physical density and ${F}_{0}$ is the fraction of the irradiated element (% by mass) in the target. Table 1 shows the volume and mass of oxygen irradiated by the four beam species (protons, helium, carbon and oxygen ions) for five samples of water plus sugar. The FWHM of the spot size for the four beam species was the one tabulated by HIT for the corresponding energy.

For the tissue surrogate inserts, we wrapped the EBT films around the inserts and irradiated the cylinders through the central longitudinal path. Those films show at which position the beam stopped.

### Energies, intensities, acquisition times, counts and statistical uncertainties

The results shown in Fig. 4a,b were obtained with an energy of 90.7 MeV and an intensity of 8 × 10^7^ p/s. The acquisition lasted 3 min (36 spills) and a total of 1.44 × 10^10^ protons were delivered. The results shown in Fig. 4c were obtained with an energy of 92 MeV/u and an intensity of 3 × 10^7^ p/s. The acquisition lasted 3 min (37 spills) and a total of 5.4 × 10^9^ helium ions were delivered. The results shown in Fig. 5 were obtained with an energy of 113.6 MeV and an intensity of 3.2 × 10^8^ p/s. The acquisition lasted 2:15 min (28 spills) and a total of 4.32 × 10^10^ protons were delivered. The results shown in Fig. 7 were obtained with an energy of 88.1 MeV/u and 161.5 MeV/u and an intensity of 8 × 10^7^ p/s and 3 × 10^7^ p/s, for helium and carbon ions, respectively. The acquisition lasted 1:41 min (21 spills) and a total of 8.14 × 10^9^ helium ions and 3.04 × 10^9^ carbon ions were delivered. The results shown in Fig. 8 were obtained with an energy of 88.1 MeV/u and an intensity of 3 × 10^8^ p/s. The acquisition lasted 1:07 min (14 spills) and a total 2.04 × 10^10^ helium ions were delivered.

The random error, Δ*N*, was calculated from the number of counts, *N*, within the 5.2 MeV peak for oxygen and the 1.66 MeV peak for calcium (Δ*N* = $\sqrt{N}$). To calculate the uncertainties in the mass of irradiated oxygen and in the oxygen and calcium concentration, we extrapolated the statistical uncertainties associated to the prompt-gamma measurements into the corresponding variable (projection of the random error into the fitted curves and from the fitted curves into the orthogonal axis).

## Supplementary information

Supplementary Information.
