# Supplementary material for: PIBS: Proton and ion beam spectroscopy for in vivo measurements of oxygen, carbon, and calcium concentrations in the human body
Source: Sci Rep. 2020 Apr 24;10:7007. doi: 10.1038/s41598-020-63215-0 (PMC7181859; doi:10.1038/s41598-020-63215-0)
Supplement: Supplementary file 1 — Supplementary Information. [file 41598_2020_63215_MOESM1_ESM.pdf]

PIBS: Proton and ion beam spectroscopy for *in vivo* measurements of oxygen, carbon, and calcium concentrations in the human body

Paulo Magalhaes Martins[1,2], Riccardo Dal Bello[1,3], Benjamin Ackermann[4],

Stephan Brons[4], German Hermann[5], Thomas Kihm[5] & Joao Seco[1,3]

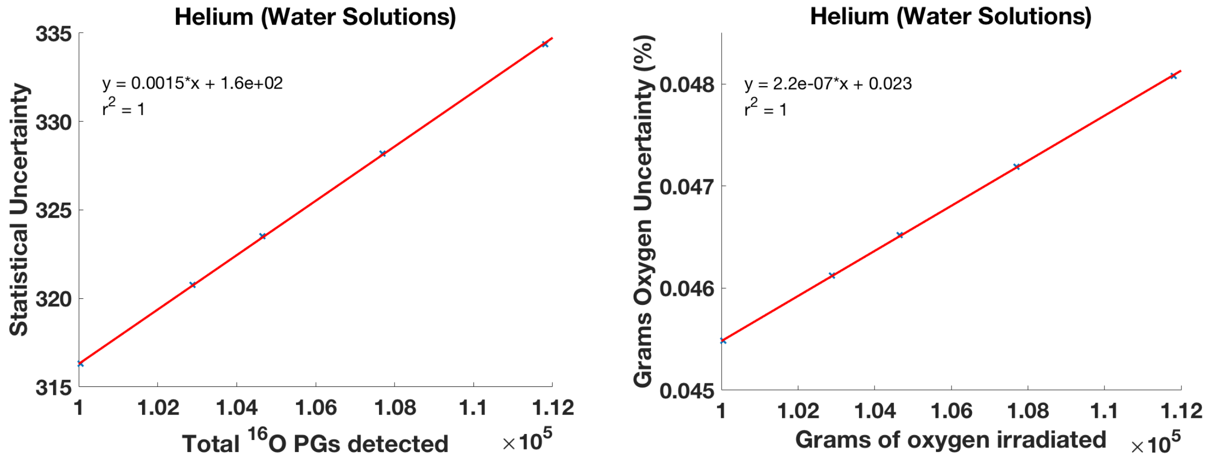

a) b)

Figure S1: **Linear relationship between the total number of prompt gammas (PGs) detected within the 5.2 MeV peak resulting from the irradiation of the five water samples by helium beams and the measured grams of oxygen irradiated.** **a,** The average statistical uncertainty, $\Delta N = 325$ counts. **b,** The average oxygen mass uncertainty, $\Delta m_O = 46.7$ mg. The average total counts per run, N = 2.6 × 10^6. From Fig. 4 (d).

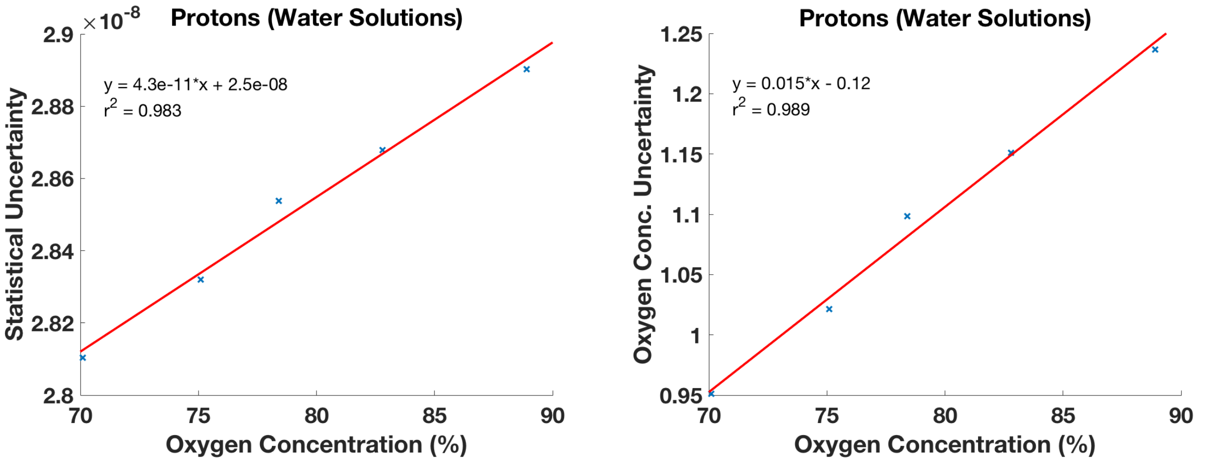

a) b)

Figure S2: **Logarithmic trend between the oxygen concentration and the prompt gamma production resulting from the irradiation of the five water samples by proton beams .** **a,** The average statistical uncertainty is 2.85 × 10^{-8}. **b,** The uncertainty in the oxygen concentration ranges from $\Delta[O] = 0.95\%$ ([O]=70.1%) to $\Delta[O] = 1.24\%$ ([O]=88.9%). The average total counts per run, N = 7.5–8 × 10^6. From Fig. 5(c).

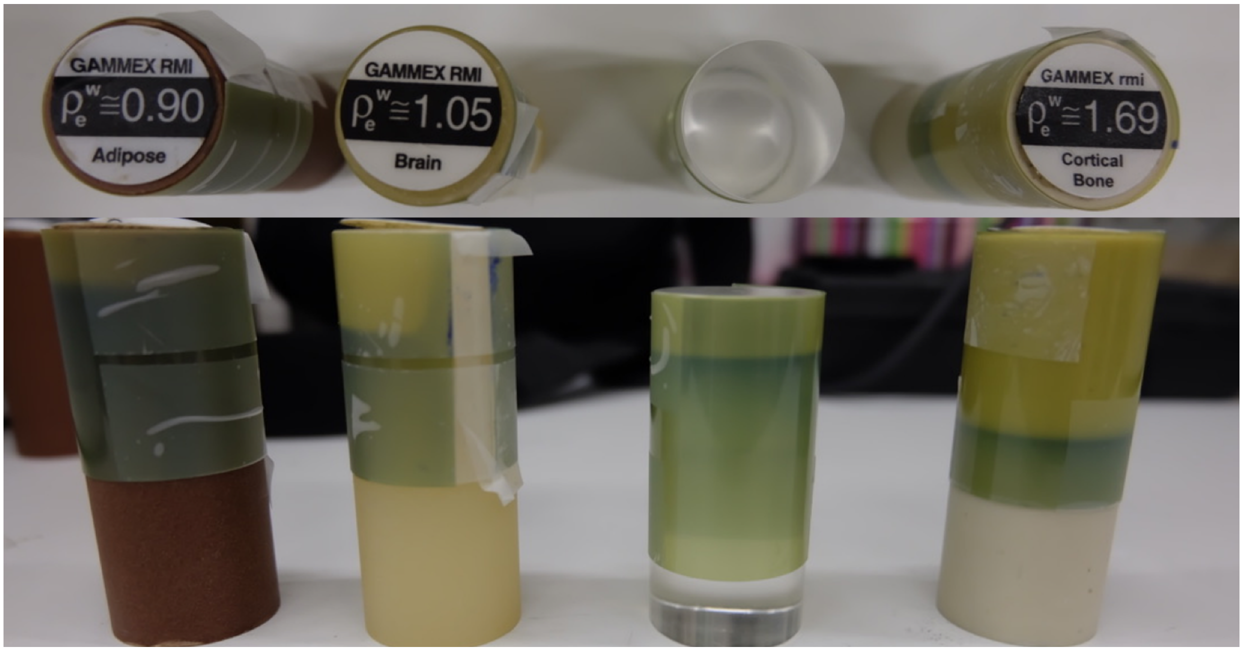

Figure S3: **Experimental setup to compare the oxygen and calcium concentration from the samples of water plus sugar and the tissue surrogate inserts. a**, Photo of the top and side view of four tissue surrogate inserts (adipose, brain, PMMA, and cortical bone) wrapped in EBT film. This zoomed image clearly shows the effect of the increasing density of the inserts on the shorter path length travelled by the particles. The beam coming from the bottom travels a shorter path length for denser materials. From Fig. 6(c).

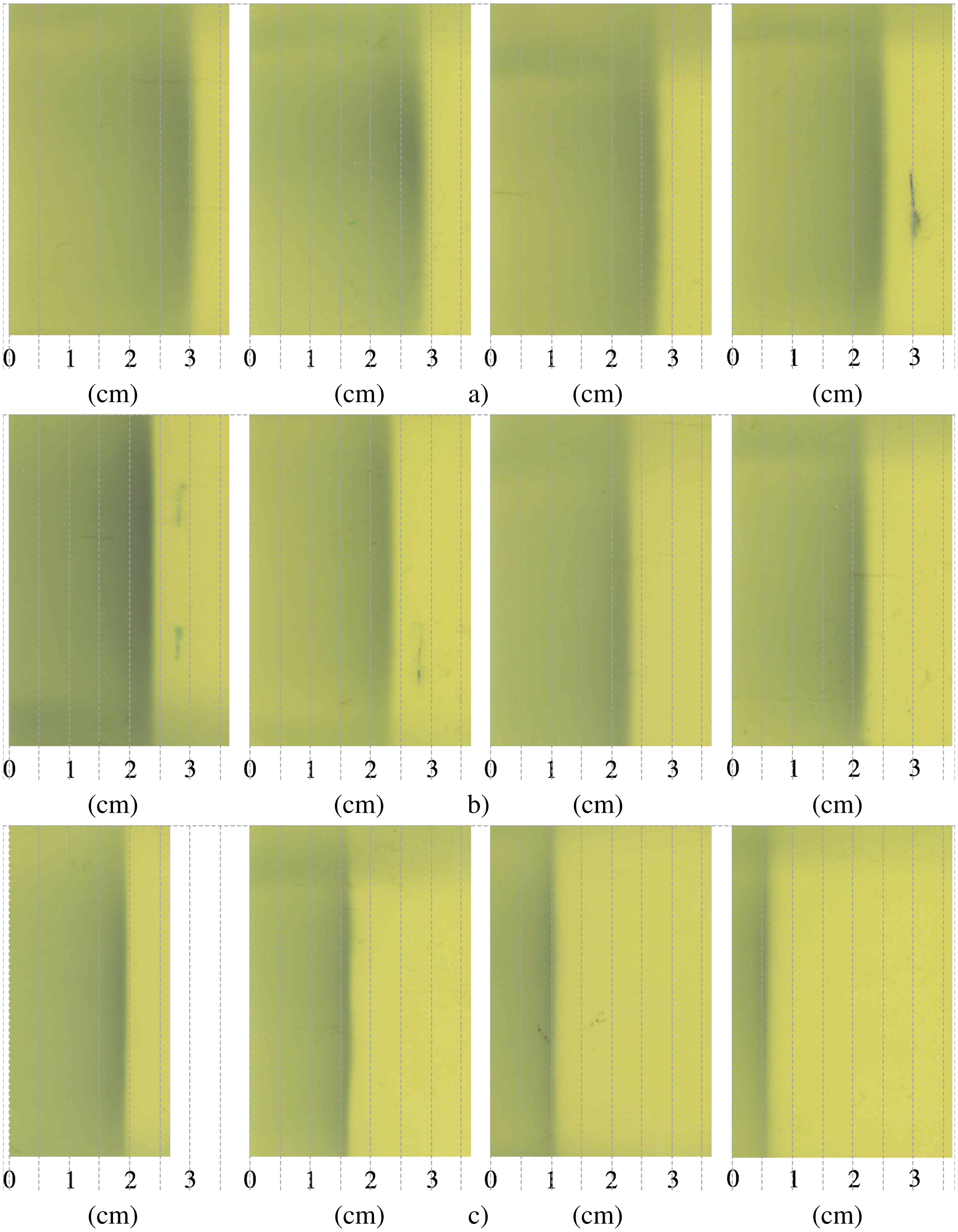

Figure S4: **EBT films wrapped around the tissue surrogates and irradiated by helium through the axis of the cylinders.** The films are ordered according to the density of the tissue surrogates from left to right and from top to bottom. **a**, adipose, breast, solid water and muscle. **b**, brain, liver, inner bone, B200-bone. **c**, PMMA, CB2-30%, CB2-50%, cortical bone. The irradiated distance = EBT irradiated film (cm) + 3.34 cm (no film). From Fig. 6(c).

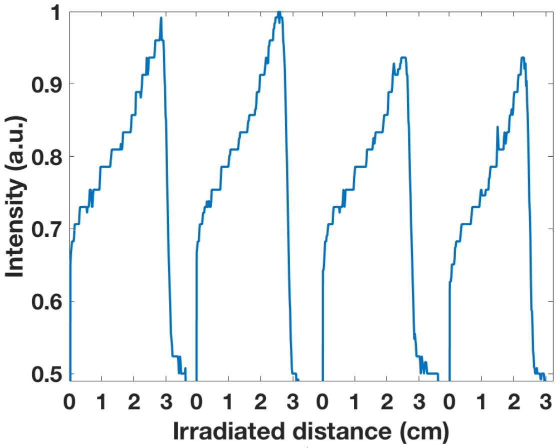

a)

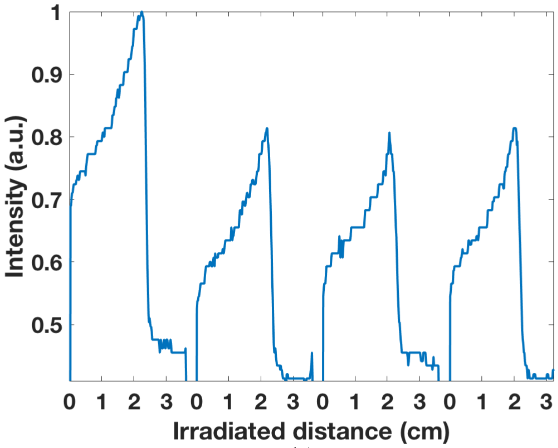

b)

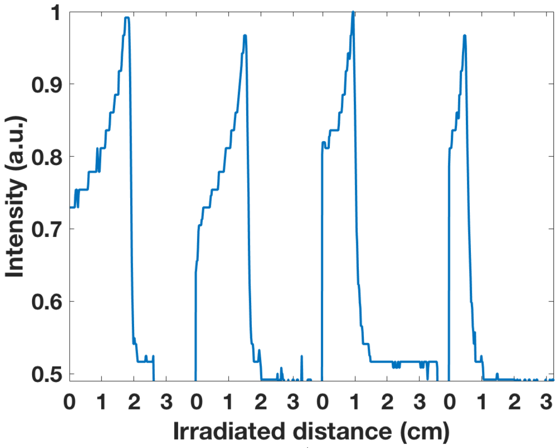

c)

Figure S5: **Profiles along the EBT films wrapped around the tissue surrogates and irradiated by helium through the axis of the cylinders. a,** adipose, breast, solid water and muscle. **b**, brain, liver, inner bone, B200-bone. **c**, PMMA, CB2-30%, CB2-50%, cortical bone. From Fig. S4.

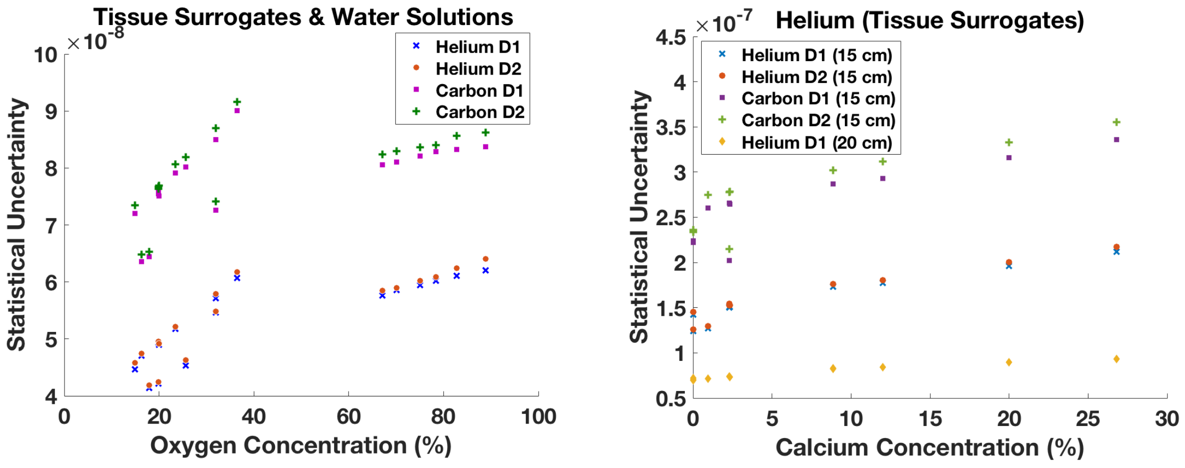

a) b)

Figure S6: **Relationship between the oxygen and calcium concentration and the prompt gamma production.** **a,** The statistical uncertainties in the measurements of the oxygen concentration range from 4.14 $\times$ 10^{-8} for helium ions to 9.16 $\times$ 10^{-8} for carbon ions. **b,** The statistical uncertainties in the measurements of the calcium concentration range from 6.97 $\times$ 10^{-8} for helium ions to 3.55 $\times$ 10^{-7} for carbon ions. From Fig. 7 (c) and Fig. 8 (d).

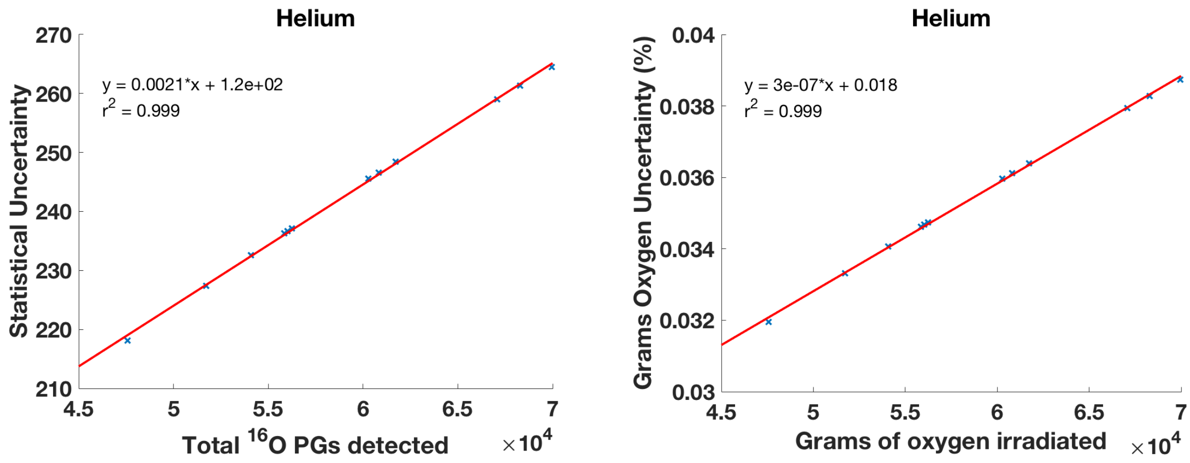

a) b)

Figure S7: **Linear relationship between the total number of prompt gammas (PGs) detected within the 5.2 MeV peak resulting from the irradiation of the tissue surrogate inserts by helium beams and the measured grams of oxygen irradiated.** **a,** The average statistical uncertainty, ΔN = 243 counts. **b,** The average oxygen mass uncertainty, Δm_O = 35.6 mg. The average total counts per run, N, ranged from 2.5 $\times 10^6$ (14 spills and brain surrogate) to 3.4 $\times 10^6$ (14 Spills and cortical bone surrogate). From Fig. 8 (b).

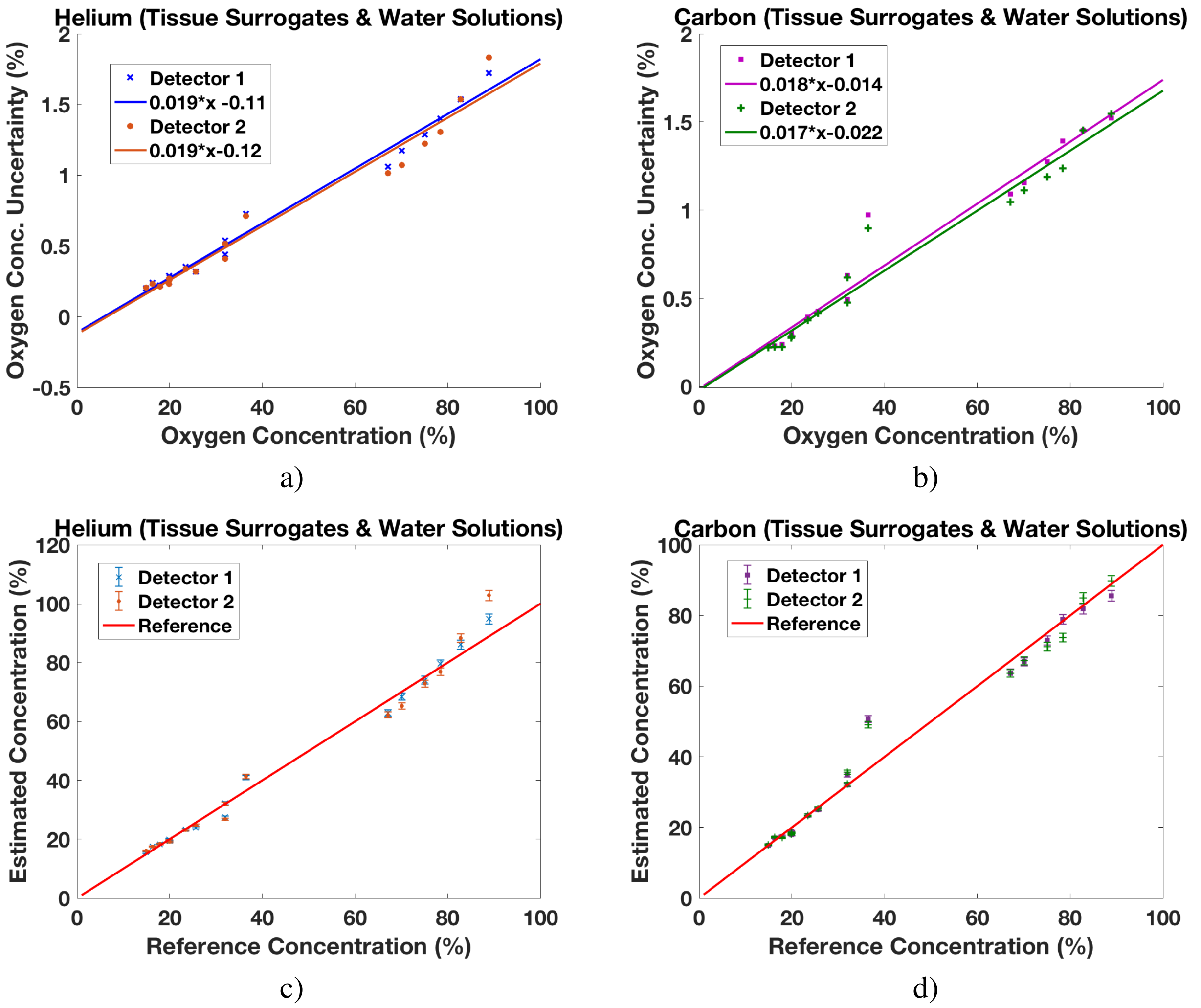

Figure S8: **Relationship between the oxygen concentration and the prompt gamma production. a–b**, Oxygen concentration uncertainties extrapolated from the error in the prompt-gamma counts both for the helium and carbon irradiation of the water solutions and the tissue surrogates with the setup with two detectors. The uncertainty in the oxygen concentration ranges from $\Delta[O] = 0.20\%$ ([O]=14.9%) to $\Delta[O] = 1.83\%$ ([O]=88.9%) for helium ions and from $\Delta[O] = 0.22\%$ ([O]=14.9%) to $\Delta[O] = 1.55\%$ ([O]=88.9%) for carbon ions. **c-d**, Estimated oxygen concentration extrapolated from the prompt-gamma counts in the same previous conditions. A reference line in red is also shown. For helium ions, the average total counts per run, N, ranged from 1.7 $\times 10^6$ (21 spills and liver surrogate) to 2.9 $\times 10^6$ (28 Spills and Water + 130 g Sugar). For carbon ions, the average total counts per run, N, ranged from 1.6 $\times 10^6$ (21 spills and brain surrogate) to 2.6 $\times 10^6$ (28 Spills and Water + 130g Sugar). From Fig. 7 (c)

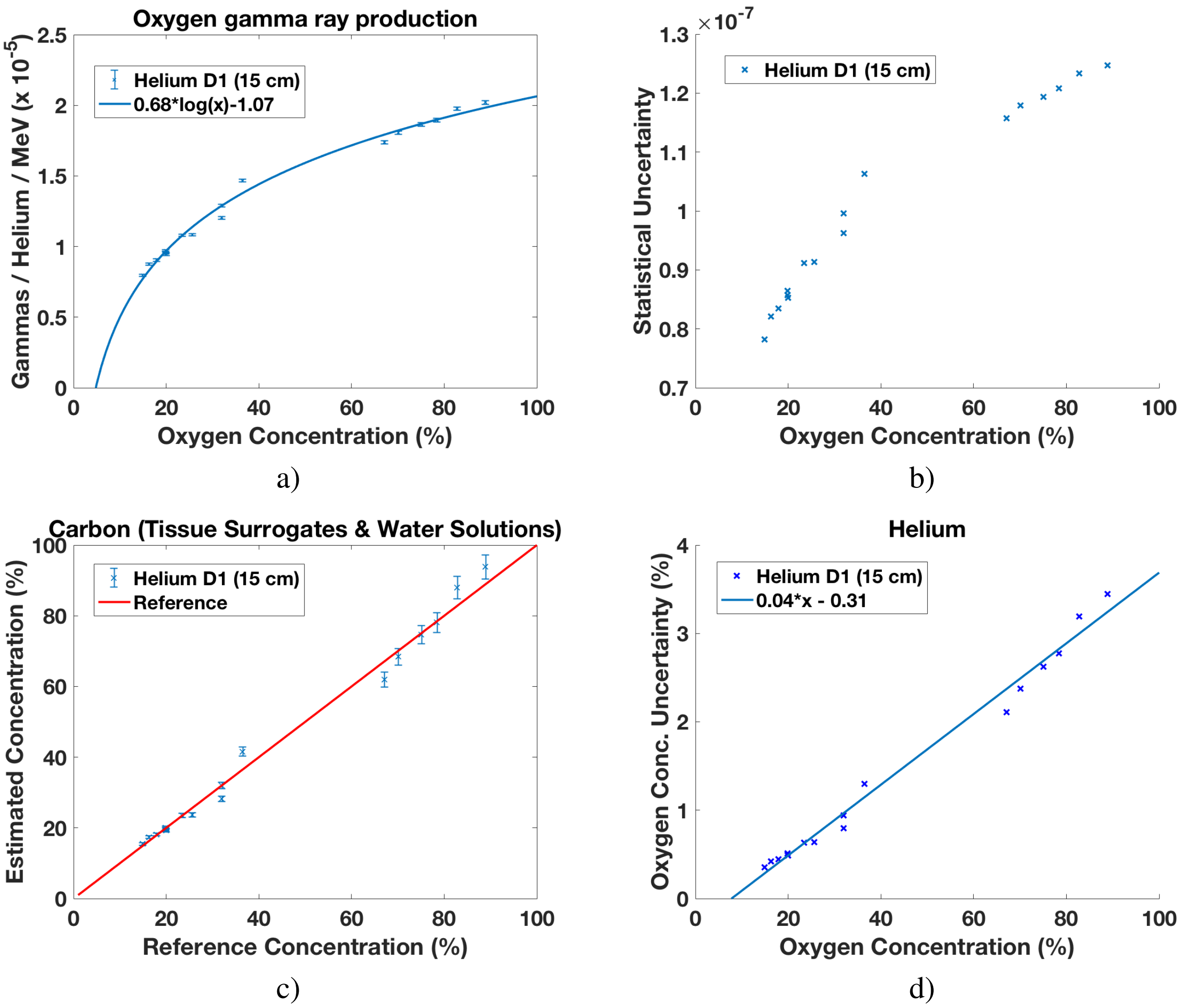

Figure S9: **Relationship between the oxygen concentration and the prompt gamma production from the irradiation by helium ions during 7 spills (33.93 s).** **a**, The data from the irradiation of the samples of water plus sugar with higher oxygen concentration and the data from the irradiation of the tissue surrogate inserts with lower oxygen concentration clearly fits a logarithmic trend. **b**, The statistical uncertainty in the measurements of the oxygen concentration ranges from 7.82×10^{-8} to 1.25×10^{-7}. **c**, Estimated oxygen concentration extrapolated from the prompt-gamma counts with the detector at 15 cm. A reference line in red is also shown. **d**, The uncertainty in the oxygen concentration ranges from $\Delta[O] = 0.36\%$ ([O]=14.9%) to $\Delta[O] = 3.4\%$ ([O]=88.9%). The total counts per run, N, ranged from 5.7 $\times 10^5$ (liver surrogate) to 9.3 $\times 10^5$ (cortical bone surrogate).

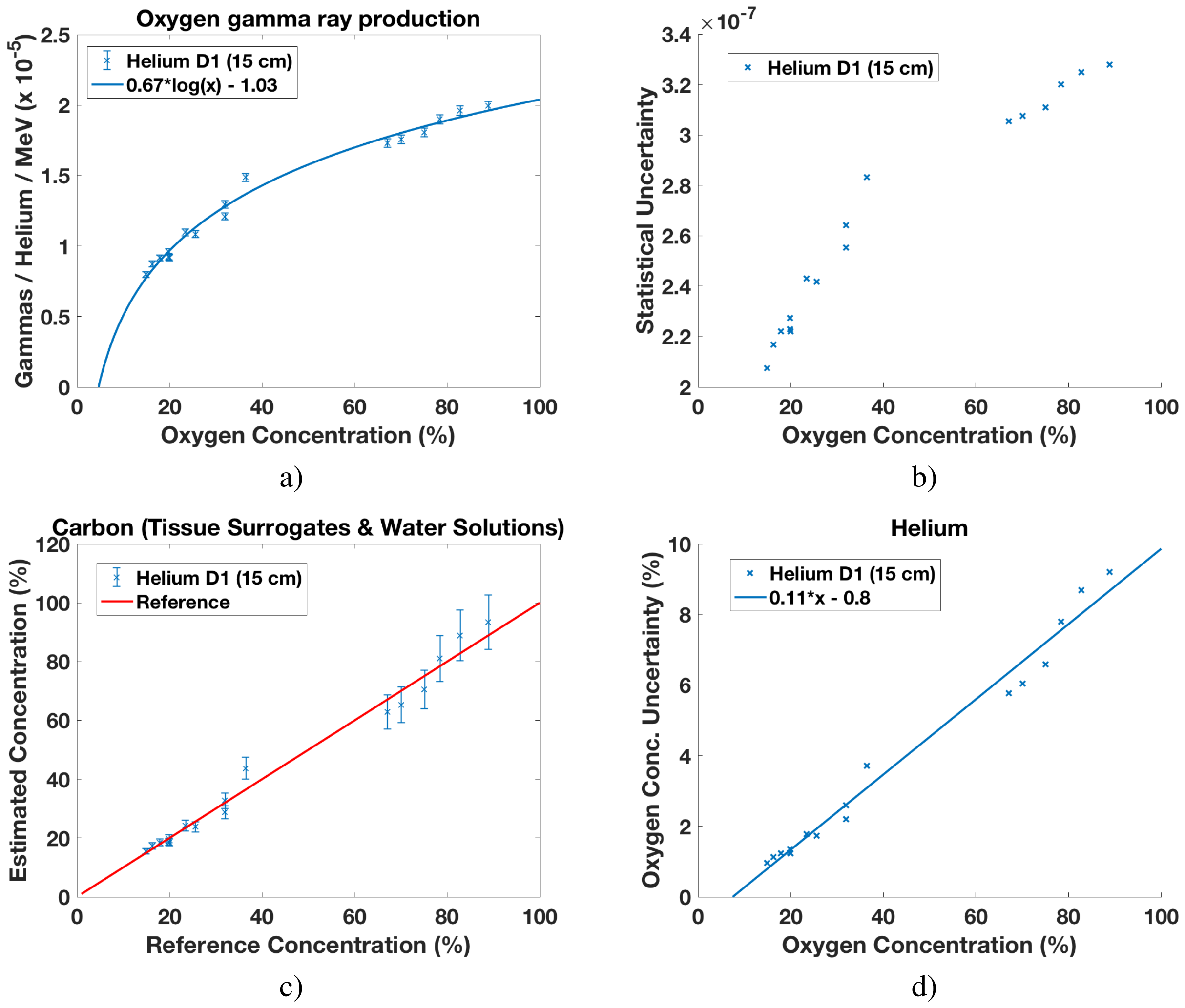

Figure S10: **Relationship between the oxygen concentration and the prompt gamma production from the irradiation by helium ions during 1 spill (4.84 s). a**, The data from the irradiation of the samples of water plus sugar with higher oxygen concentration and the data from the irradiation of the tissue surrogate inserts with lower oxygen concentration clearly fits a logarithmic trend. **b**, The statistical uncertainty in the measurements of the oxygen concentration ranges from 2.07×10^{-7} to 3.28×10^{-7}. **c**, Estimated oxygen concentration extrapolated from the prompt-gamma counts with the detector at 15 cm. A reference line in red is also shown. **d**, The uncertainty in the oxygen concentration ranges from $\Delta[O] = 0.97\%$ ([O]=14.9%) to $\Delta[O] = 9.2\%$ ([O]=88.9%). The total counts per run, N, ranged from 8.1 $\times 10^4$ (liver surrogate) to 1.2 $\times 10^5$ (cortical bone surrogate).

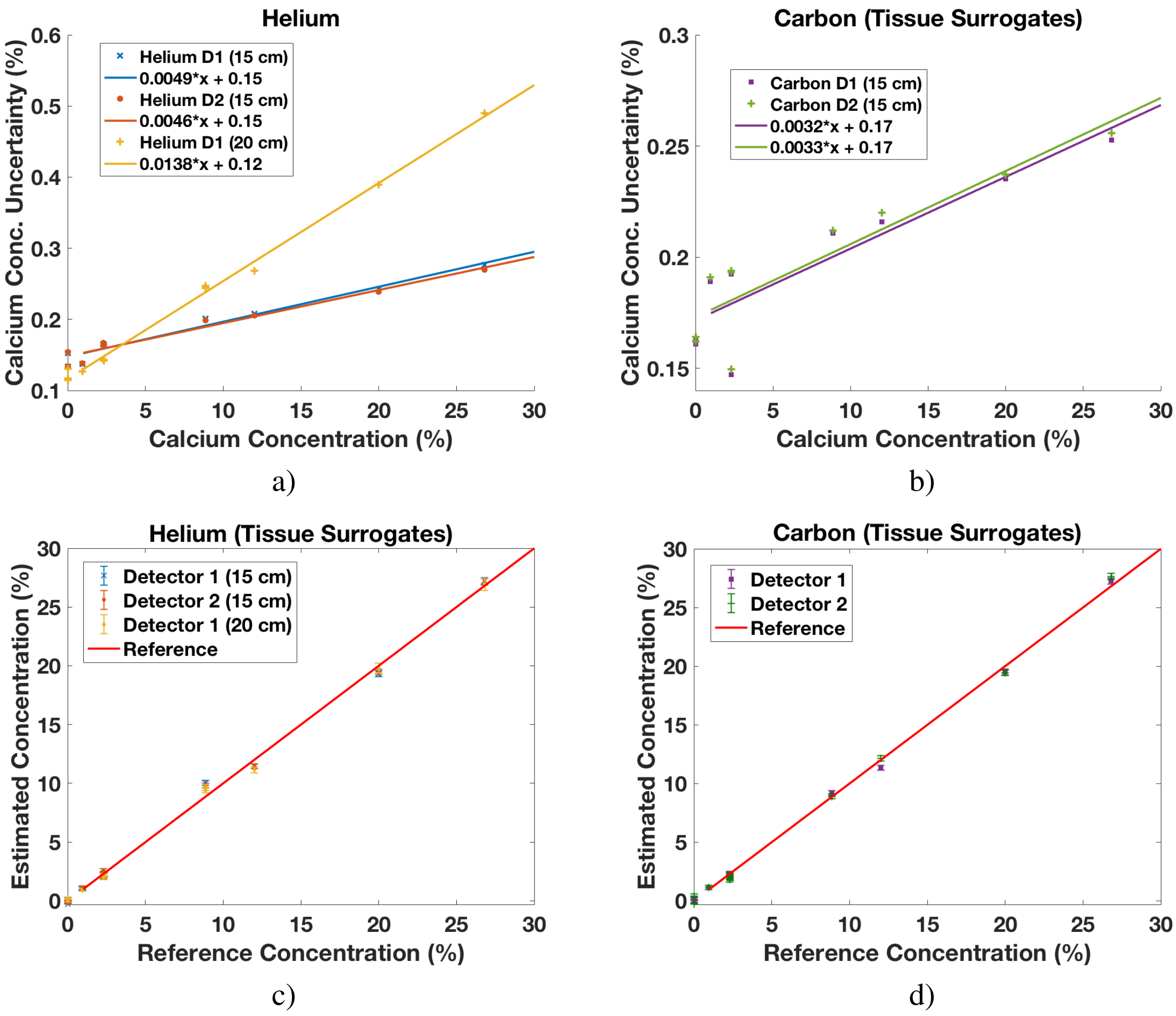

Figure S11: **Relationship between the calcium concentration and the prompt gamma production.** **a–b**, Calcium concentration uncertainties extrapolated from the error in the prompt-gamma counts both for the helium and carbon irradiation of the tissue surrogates with the setup with two detectors at 15 cm and the setup with one detector at 20 cm. The uncertainty in the calcium concentration ranges from $\Delta[Ca] = 0.14\%$ ([Ca]=0.95%) to $\Delta[Ca] = 0.28\%$ ([Ca]=26.8%) for helium ions and the detector at 15 cm. For carbon ions, it ranges from $\Delta[Ca] = 0.15\%$ ([Ca]=2.31%) to $\Delta[Ca] = 0.25\%$ ([Ca]=26.8%). **c-d**, Estimated calcium concentration extrapolated from the prompt-gamma counts in the same previous conditions. A reference line in red is also shown. From Fig. 8 (d).

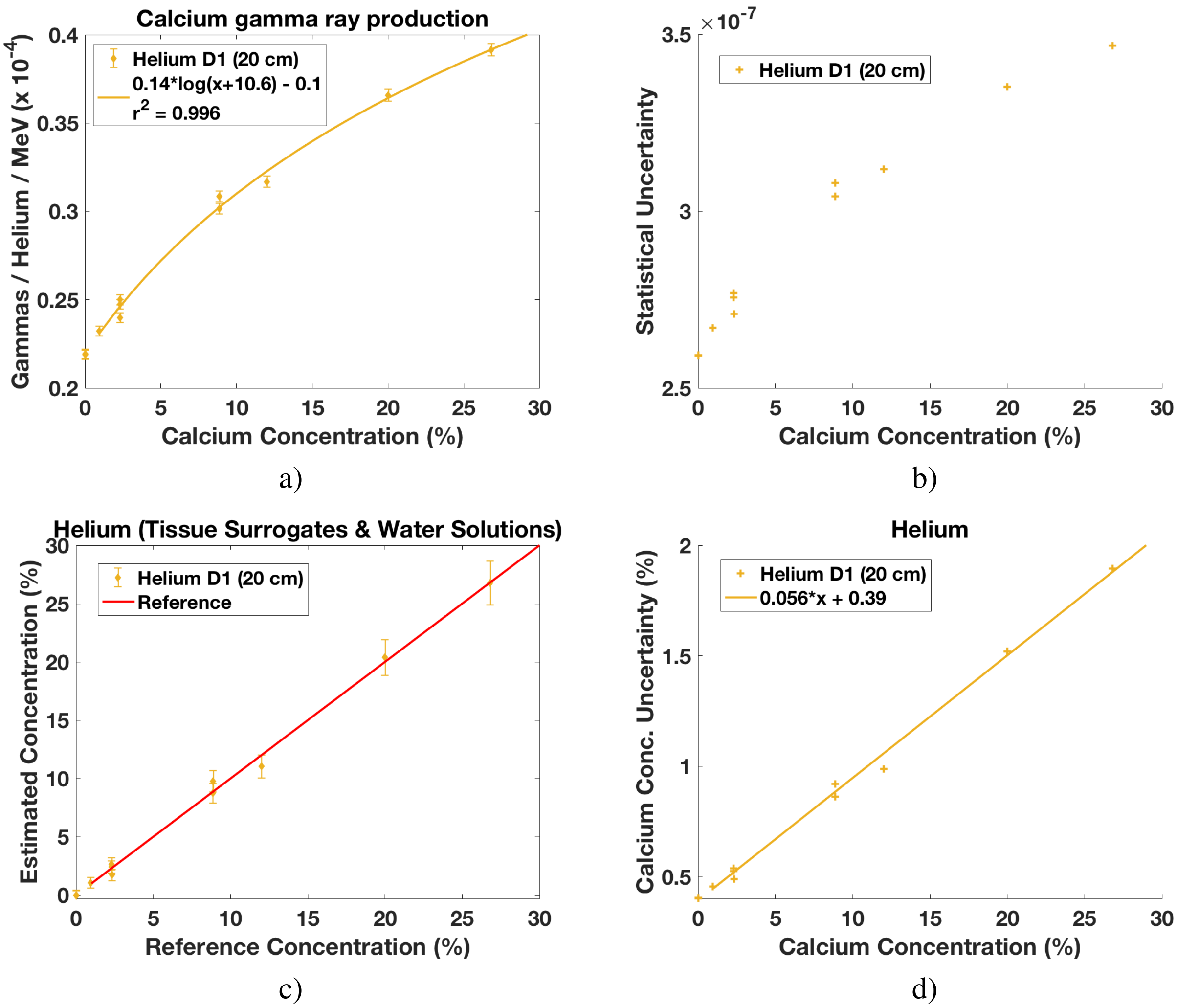

Figure S12: **Relationship between the calcium concentration and the prompt gamma production from the irradiation by helium ions during 1 spill (4.84 s).** **a**, The data from the irradiation of the tissue surrogate inserts with low calcium concentrations clearly fits a logarithmic trend. **b**, The statistical uncertainty in the measurements of the calcium concentration ranges from 2.59×10^{-7} to 3.47×10^{-7}. **c**, Estimated calcium concentration extrapolated from the prompt-gamma counts with the detector at 15 cm. A reference line in red is also shown. **d**, The uncertainty in the calcium concentration ranges from $\Delta[Ca] = 0.46\%$ ([Ca]=0.95%) to $\Delta[Ca] = 1.9\%$ ([Ca]=26.8%). The total counts per run, N, ranged from 1.8 $\times 10^5$ (brain surrogate) to 2.5 $\times 10^5$ (cortical bone surrogate).

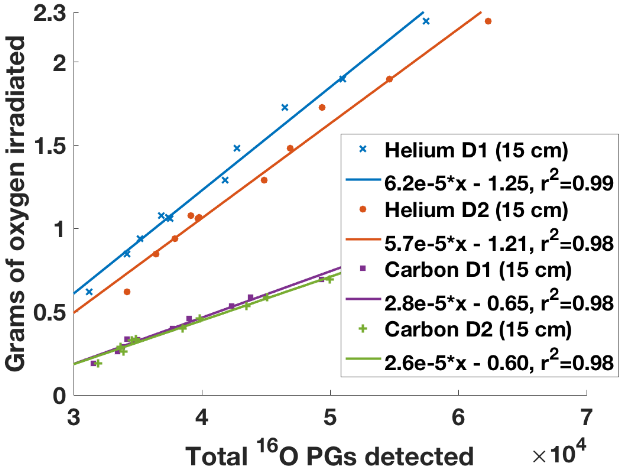

Figure S13: **Linear relationship between the total number of prompt gammas (PGs) detected within the 5.2 MeV peak resulting from the irradiation of the tissue surrogate inserts by helium and carbon beams and the measured grams of oxygen irradiated.** The measured grams of oxygen irradiated within the tissue surrogate inserts by helium and carbon beams and detected by two detectors are linearly related to the total prompt gammas (PGs) detected within the 5.2 MeV peak. This reproduces the results from Fig. 8 (b).

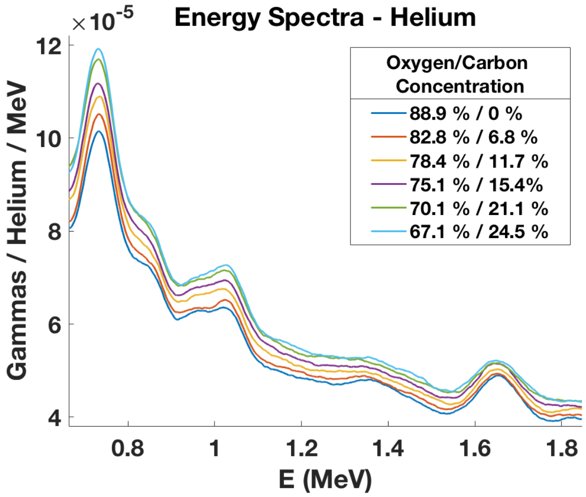

Figure S14: **Prompt gamma energy spectra resulting from the irradiation of six water solutions.** The prompt gamma energy spectra shows the increasing line at 1.6-1.7 MeV. This reaction competes with the reaction from the calcium irradiation. These spectra were obtained from the irradiation of the water solutions by helium ions with the detector 1 placed at 15 cm from the beam axis. The energy peak at 0.718 MeV has a clear increase with the carbon concentration. From Fig. 7 (a).
